# Rate-dependent phase transitions in Li_2_FeSiO_4_ cathode nanocrystals

**DOI:** 10.1038/srep08599

**Published:** 2015-02-26

**Authors:** Xia Lu, Huijing Wei, Hsien-Chieh Chiu, Raynald Gauvin, Pierre Hovington, Abdelbast Guerfi, Karim Zaghib, George P. Demopoulos

**Affiliations:** 1 Materials Engineering, McGill University, Montréal, Québec H3A 0C5, Canada; 2 Institut de recherché d′ Hydro-Québec (IREQ), Varennes, Québec J3X 1S1, Canada

## Abstract

Nanostructured lithium metal orthosilicate materials hold a lot of promise as next generation cathodes but their full potential realization is hampered by complex crystal and electrochemical behavior. In this work Li_2_FeSiO_4_ crystals are synthesized using organic-assisted precipitation method. By varying the annealing temperature different structures are obtained, namely the monoclinic phase at 400°C, the orthorhombic phase at 900°C, and a mixed phase at 700°C. The three Li_2_FeSiO_4_ crystal phases exhibit totally different charge/discharge profiles upon delithiation/lithiation. Thus the 400°C monoclinic nanocrystals exhibit initially one Li extraction via typical solid solution reaction, while the 900°C orthorhombic crystals are characterized by unacceptably high cell polarization. In the meantime the mixed phase Li_2_FeSiO_4_ crystals reveal a mixed cycling profile. We have found that the monoclinic nanocrystals undergo phase transition to orthorhombic structure resulting in significant progressive deterioration of the material's Li storage capability. By contrast, we discovered when the monoclinic nanocrystals are cycled initially at higher rate (C/20) and subsequently subjected to low rate (C/50) cycling the material's intercalation performance is stabilized. The discovered rate-dependent electrochemically-induced phase transition and stabilization of lithium metal silicate structure provides a novel and potentially rewarding avenue towards the development of high capacity Li-ion cathodes.

Li-ion batteries (LIBs) are omnipresent in everyday life powering the portable electronics to which consumers have come to depend. Now LIBs are called to power the plug-in hybrid (PHEV) and electric vehicles (EV) as our society is moving towards electrification of transportation to address the serious global issue of climate change^1^,^2^,^3^,^4^. Automotive LIBs require high-energy (and power) densities without compromising safety, long lifetime, or economics. It is recognized that for the development of higher energy density LIBs the main limitation is the cathode material in terms of capacity and voltage^5^,^6^,^7^. Among the currently developed cathode materials, such as layered transition metal oxides Li**M**O_2_ (*M* = Co, Mn, Ni, *etc.*), spinel LiMn_2_O_4_, and the LiFePO_4_ olivine (LFP), the latter one stands out for its remarkable thermal safety endowed by the inductive effect of the phosphate polyanion^8^,^9^,^10^. However for next generation LIB-powered vehicles, development of polyanion cathode materials with higher energy density than LFP cathode as is the case of silicates, Li_2_**M**SiO_4_ (**M** = Fe, Mn or Co, *etc.*) is sought^11^,^12^,^13^,^14^,^15^,^16^,^17^,^18^,^19^,^20^. The theoretical specific capacity of Li_2_**M**SiO_4_ is as large as twice (330 mAh/g) that of olivine LiFePO_4_, hence the great potential and opportunity.

Li_2_**M**SiO_4_ compounds belong to the tetrahedral structure material family known to exhibit several temperature-dependent polymorphs, namely high temperature monoclinic (γ_0_, *P2_1_/n*) and orthorhombic (*γ_II_, Pmnb*) and low temperature orthorhombic (*β_II_, Pmn2_1_*) which can significantly influence/determine electrochemical cycling behavior^21^,^22^. Presently most of the studies report successful results only with the first Li insertion/extraction step with considerable capacity loss upon further cycling due to phase instability^16^,^23^. In addition due to very poor intrinsic electronic/ionic conductivity^23^, the capacity of Li_2_**M**SiO_4_ materials declines dramatically with the increase of rate and/or the operation potential. This has prompted researchers into various ways to enhance the Li^+^/*e*^−^ transport properties of silicates, such as size reduction and nanostructuring, carbon coating and hierarchical structures^24^,^25^,^26^,^27^. However for unlocking the full capacity potential of the silicates the underlying Li storage, transport and phase transition phenomena need to be systematically understood and ultimately controlled. This study indeed is contributing to this need by reporting on a less explored behavior of silicates that of rate-dependent phase transition in Li_2_FeSiO_4_.

Concerning the Li storage and transport mechanism in Li_2_MSiO_4_, Nyten *et al.* demonstrated that the observed lowering of the potential plateau from 3.10 to 2.80 V during the first cycle of Li_2_FeSiO_4_ particles can be explained by a structural rearrangement in which some of the Li ions (in the *4b* site) and Fe ions (in the *2a* site) become interchanged^13^. Subsequent to that Kuganathan and Islam revealed using first-principle simulations that the Li-M exchange positions is the most energy favorable intrinsic defect in both monoclinic and orthorhombic Li_2_MnSiO_4_ structures^28^. On the basis of these simulations the Li_2_**M**SiO_4_ structures should experience severe cation rearrangements upon cycling. Along these lines Armstrong *et al.* reported conversion of monoclinic (*γ_0_*) to orthorhombic (*β_II_*) upon initial cycling of Li_2_FeSiO_4_ at 50°C and C/16 (1C = 160 mA/g)^21^ as did Chen *et al.* at room temperature and C/20^29^. However, studies by Lv *et al.* and Ferrari *et al.*, who charged/discharged their monoclinic Li_2_FeSiO_4_ at higher rate, C/10 to C/8 (1C = 160 mA/g) revealed instead the formation of a disordered (antisite defect) monoclinic phase^30^,^31^. In the meantime, Masese *et al.* have just reported that at C/50 rate initial lithium extraction leads to complete transformation from monoclinic Li_2_FeSiO_4_ to a thermodynamically stable orthorhombic LiFeSiO_4_ structure, accompanied with the occurrence of significant Li/Fe antisite mixing; but at C/10 rate of lithium extraction and insertion the parent monoclinic Li_2_FeSiO_4_ phase is retained (as metastable) with little cationic mixing^32^. The above apparent contradictory results point to a complex structure-electrochemistry relationship, when it comes to the type of phase transitions experienced by silicate cathode materials at different charging/discharging rates. Elucidating and ultimately controlling this relationship constitutes the key in realizing the full reversible capacity of silicates enabling thus the making of safe Li-ion batteries with high specific energy to power the next generation of electric vehicles.

In this work, different (monoclinic, orthorhombic and mixed phases) Li_2_FeSiO_4_ crystalline polymorphs are prepared using organic-assisted hydrothermal precipitation and annealing at different temperatures (400°C, 700°C and 900°C). The structure and electrochemical response of all three phases are investigated comparatively for the first time. Previous electrochemical works have focused only on the high temperature monoclinic (*γ_0_*, *P2_1_/n*) Li_2_FeSiO_4_ (LFS) as pristine cathode material. The galvanostatic charging/discharging of the orthorhombic (*γ_II_, Pmnb*) LFS has not been reported previously as starting cathode material. The different silicate phase particles are not subjected to carbon coating to allow for direct probing of their phase transition behavior during galvanostatic delithiation (charging)/lithiation (discharging). The results demonstrate that at very slow rate (C/50, 1C = 165 mA/g) the monoclinic Li_2_FeSiO_4_ nanoparticles exchange initially 1 Li while the orthorhombic Li_2_FeSiO_4_ materials < 0.4 Li. Gradually however the monoclinic phase undergoes capacity fading eventually assuming the electrochemical characteristics of the orthorhombic phase. In contrast, we have discovered that prior galvanostatic charging/discharging at higher rate (C/20) helps stabilize the monoclinic phase leading to capacity retention without evidence of transition towards the orthorhombic structure after switching the rate back to the rate of C/50.

## Results and discussion

### Characterization

**Figure 1:**
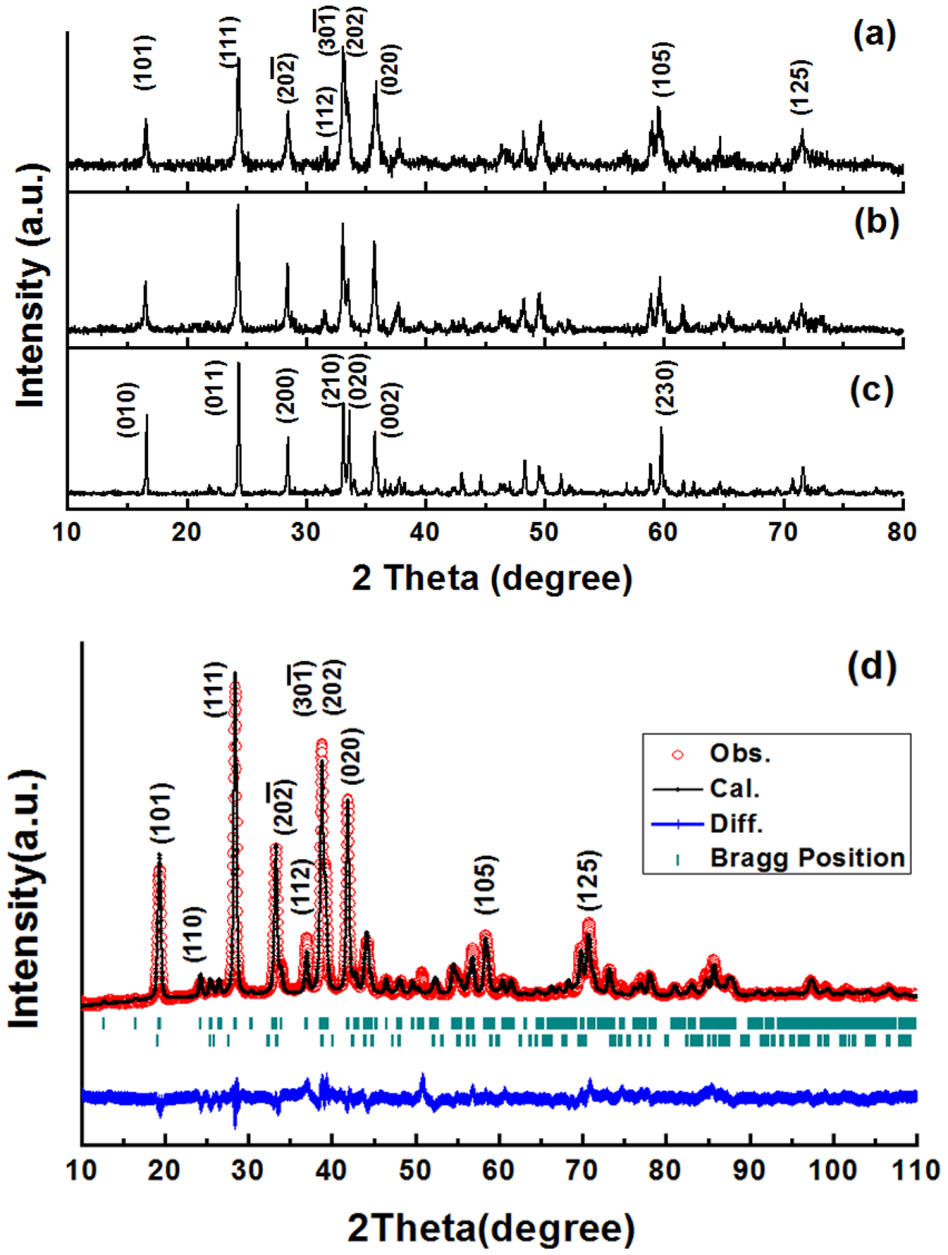
XRD patterns for the as-prepared Li_2_FeSiO_4_ samples. Li_2_FeSiO_4_ annealed at (a) 400°C, (b) 700°C and (c) 900°C using Cu K_α_ radiation (λ ~ 1.54056 Å) source. (d) The XRD pattern for the 700°C Li_2_FeSiO_4_ sample using Co K_α_ radiation (λ ~ 1.78892 A) was refined with two phases (monoclinic *P2_1_* and orthorhombic *Pmn2_1_*) with R_p _ = 4.17, R_wp_ = 5.58 and R_exp_ = 2.89 (not corrected for background) by Fullprof software.

Lithium metal orthosilicates (Li_2_**M**SiO_4_) can be categorized into two different crystal families, namely monoclinic phase and orthorhombic phase for which different space groups have been proposed to describe the atomic occupations (*P1, P2_1_* and *P2_1_/n* for monoclinic phase and *Pmn2_1_*, *Pmnb* for orthorhombic phase)^28^,^33^,^34^. The 400°C, 700°C and 900°C synthesized Li_2_FeSiO_4_ materials are denoted as LFS@400, LFS@700 and LFS@900 hereafter; their color was respectively black, dark grey and white. Figure 1 shows the XRD patterns of the as-prepared Li_2_FeSiO_4_ materials after annealing at different temperature. The XRD pattern of LFS@400 displays the widest full width at half maximum (FWHM) while the LFS@900 has the narrowest one reflecting a shift from nanocrystal to microcrystal domain caused by the elevation of the annealing temperature. In Figure 1a and 1c using Cu K_α_ radiation (λ ~ 1.54056 Å) source, the XRD peaks of the LFS@400 and LFS@900 samples can be correctly assigned to the monoclinic and orthorhombic phases, respectively. The differentiating features between the two structures lie in: 1) the existence/absence of the peak locating at *ca.* 31.6°, corresponding to the (112) plane of monoclinic structure; 2) the intensity ratio of the peaks located at *ca.* 33.1°/33.6°, corresponding to the 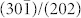 planes in monoclinic phase (*P2_1_* phase) and the (210)/(020) planes in orthorhombic phase (*Pmn2_1_* phase), which is larger in the case of well crystallized monoclinic phase^35^. In the case of the LFS@700 sample, according to Figure 1b its XRD pattern may be assigned into the monoclinic phase if the presence of the (112) peak is taken as evidence for that using Cu K_α_ radiation (λ ~ 1.54056 Å) source. However the peak intensity is not strong enough as that in perfect monoclinic structure. After structure refinement as shown in Figure 1d, it is revealed that the best results for the LFS@700 material corresponds to coexistence of the monoclinic with the orthorhombic phase at a ratio of 90/10 using Co K_α_ radiation (λ ~ 1.78892 A) source. The refined structure parameters are, *a* = 8.21(5) Å, *b* = 5.01 (0) Å, *c* = 8.22 (8) Å and α = γ = 90°, β = 99.10 (3)° for the LFS monoclinic (*P2_1_*) phase and *a* = 6.27 (0) Å, *b* = 5.42 (4) Å, *c* = 5.20(4) Å and α = γ = β = 90° for the LFS orthorhombic (*Pmn2_1_*) phase, which are in consistence with the reported values elsewhere^33^,^34^.

Besides checking for phase coexistence, the materials were checked also for impurities, an important issue in lithium metal orthorsilicate synthesis. As per Figure S1a, there is significant peak overlap between two commonly encountered iron oxides (hematite-Fe_2_O_3_ and Fe_0.95_O)^36^,^37^ and the as-prepared LFS@400 sample. Beyond the overlapping peaks, however there are secondary distinct peaks of the oxides (*e.g.* ~ 54° of hematite phase in Figure S1a) that are not present in the XRD pattern of the LFS@400 material suggesting that the latter is essentially impurity-free. Another common impurity is lithium silicate (Li_2_SiO_3_). As shown in Figure S1b this impurity was detected to be present in some of the LFS@900 samples, however fortunately it is electrochemically inactive not interfering with the testing of LFS as cathode. When comparing the XRD patterns of LFS@900 in Figure 1c and Figure S1b, the characteristic XRD peaks of lithium silicate are absent in Figure 1c, indicating that the LFS@900 sample used in subsequent electrochemical tests was phase-pure.

**Figure 2:**
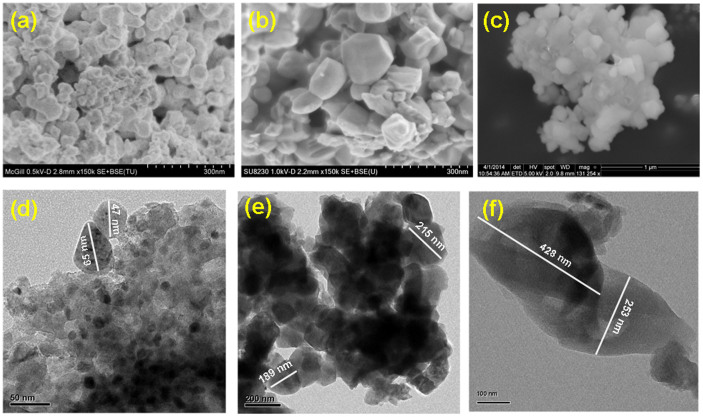
SEM, TEM morphologies of the as-prepared Li_2_FeSiO_4_ samples. (a)&(d), (b)&(e) and (c)&(f) correspond to the SEM and TEM morphologies on the Li_2_FeSiO_4_ samples annealed at 400°C, 700°C and 900°C, respectively.

Figure 2 shows SEM and TEM morphological features of the different phase LFS samples. The morphology of the LFS@400 monoclinic material is in the form of *ca.* 50 nm size nanograined beads as shown in Figure 2a and 2d. Furthermore the nanobeads form larger porous agglomerated clusters (Figure S2a). BET analysis (data in Figure S2d and Table S1) revealed indeed the LFS@400 material to be mesoporous as evident by its Type II isotherm (Figure S2d) having average pore size 17 nm (data given in Table S2) and specific surface area 28.10 m^2^/g corresponding to ~ 67 nm equivalent spherical particle size. This mesoporous structure facilitates electrolyte infiltration providing increased contact surface area that is beneficial for Li ion diffusion. The morphology of the LFS@700 material (specific surface area of 4.19 m^2^/g) maintains the mesoporous structure but this time the crystal size has increased to about 180 nm as shown in Figure 2b, 2e and Figure S2b. However the orthorhombic material obtained after annealing at 900°C (1.66 m^2^/g) is seen to be made of dense significantly enlarged (about 350 nm) particles as shown in Figure 2c, 2f and Figure S2c. Obviously, the LFS crystals grow bigger and become denser with increasing annealing temperature.

**Figure 3:**
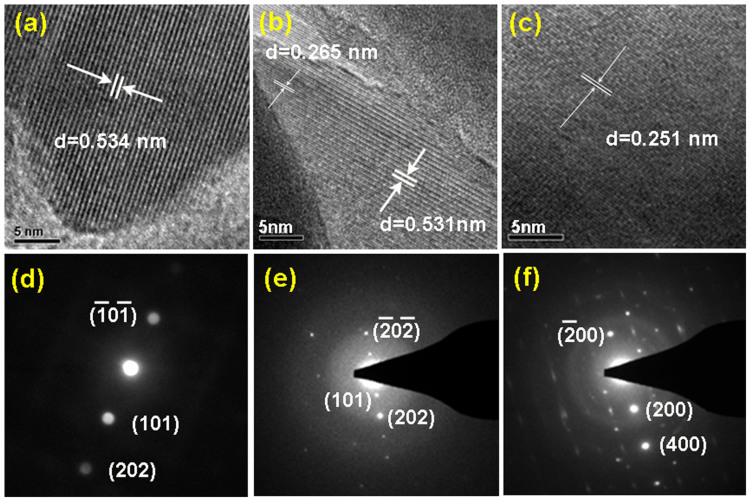
TEM crystal structure characterization of Li_2_FeSiO_4_ samples. (a)&(d), (b)&(e) and (c)&(f) correspond to the Li_2_FeSiO_4_ samples annealed at 400°C, 700°C at 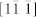 zone axis and 900°C at [100] zone axis, respectively.

The structure of three orthosilicate materials is further probed with the TEM/SAED data presented in Figure 3 and in Figure S3. As it can be seen in Figure 3a at 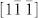 zone axis, the lattice stripes give birth to a layer distance of *d* = 0.534 nm, corresponding to the (101) plane of the monoclinic LFS (also shown in Figure S1d). The monoclinic nanostructure of the LFS@400 material is also confirmed by the SAED shown in Figure 3d and Figure S3b. Crystallographically, the monoclinic phase exhibits a layered structure with the Li ions distributed between the two Fe(Si)O_4_ slabs at 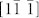 zone axis. This layered structure favors Li ion diffusion inside the (101) plane rather than going through the Fe(Si)O_4_ slabs as determined by theoretical simulations^36^. The image acquired with the layer distance of *d* = 0.360 nm can be obviously assigned to the (111) plane of the monoclinic phase as shown in Figure S3c.

With reference to the mixed phase of LFS@700 material, the following observations are made: 1) a layer distance of *d* = 0.531 nm is measured inside the crystal, which corresponds to the (101) face while a layer distance of *d* = 0.265 nm is obtained at the topmost surface layer, corresponding to the (202) face of the monoclinic phase as evidenced in Figure 3b, 3e and Figure S1c. These structural features imply that the monoclinic crystals grow layer by layer with the addition of Fe(Si)O_4_ slabs perpendicular to the (101) face, the latter face possibly being the energetically favorable lattice plane of the monoclinic phase. In addition to the predominantly monoclinic phase features the LFS@700 material exhibited (as detected by TEM-see Figure S4a) a layer distance *d* = 0.366 nm that is consistent with the (020) face of the orthorhombic LFS phase. The presence of orthorhombic crystals along the monoclinic crystals in LFS@700 is also evidenced by the SAED pattern shown in Figure S4b. Hence the TEM/SAED data confirm the mixed phase composition of the LFS@700 material that was determined by XRD refinement. The two phases occupy different nano-areas, like a grain-by-grain nanodomain structure with some interesting/important two-phase boundary, which might play an important role in the electrochemically induced phase transitions as discussed later. The orthorhombic character of LFS@900 material is clearly evident in Figure 3c and 3f, where the layer distance of *d* = 0.251 nm corresponds to the (002) face at [100] zone axis. The SAED diffraction pattern in Figure 3f exhibits however intermittent diffraction lines, which is indicative of the existence of structure defects (possibly due to random LiO_4_ tetrahedra distributions) in planes perpendicular to the *a* axis^34^.

As per phase characterization presented above for the three annealed LFS materials it becomes evident that kinetically the formation of the monoclinic phase is favored during conversion of the original precursor obtained after hydrothermal treatment. Thus at 400°C the material has crystallized in the monoclinic phase made of mesoporous nanocrystals. Upon elevation of the annealing temperature to 700°C although the material remains predominantly monoclinic we observe the appearance of the thermodynamically favored orthorhombic phase. Conversion of the monoclinic to orthothombic is possibly completed at 900°C. The specific mechanism of phase transformations during annealing is the subject of further investigation.

**Figure 4:**
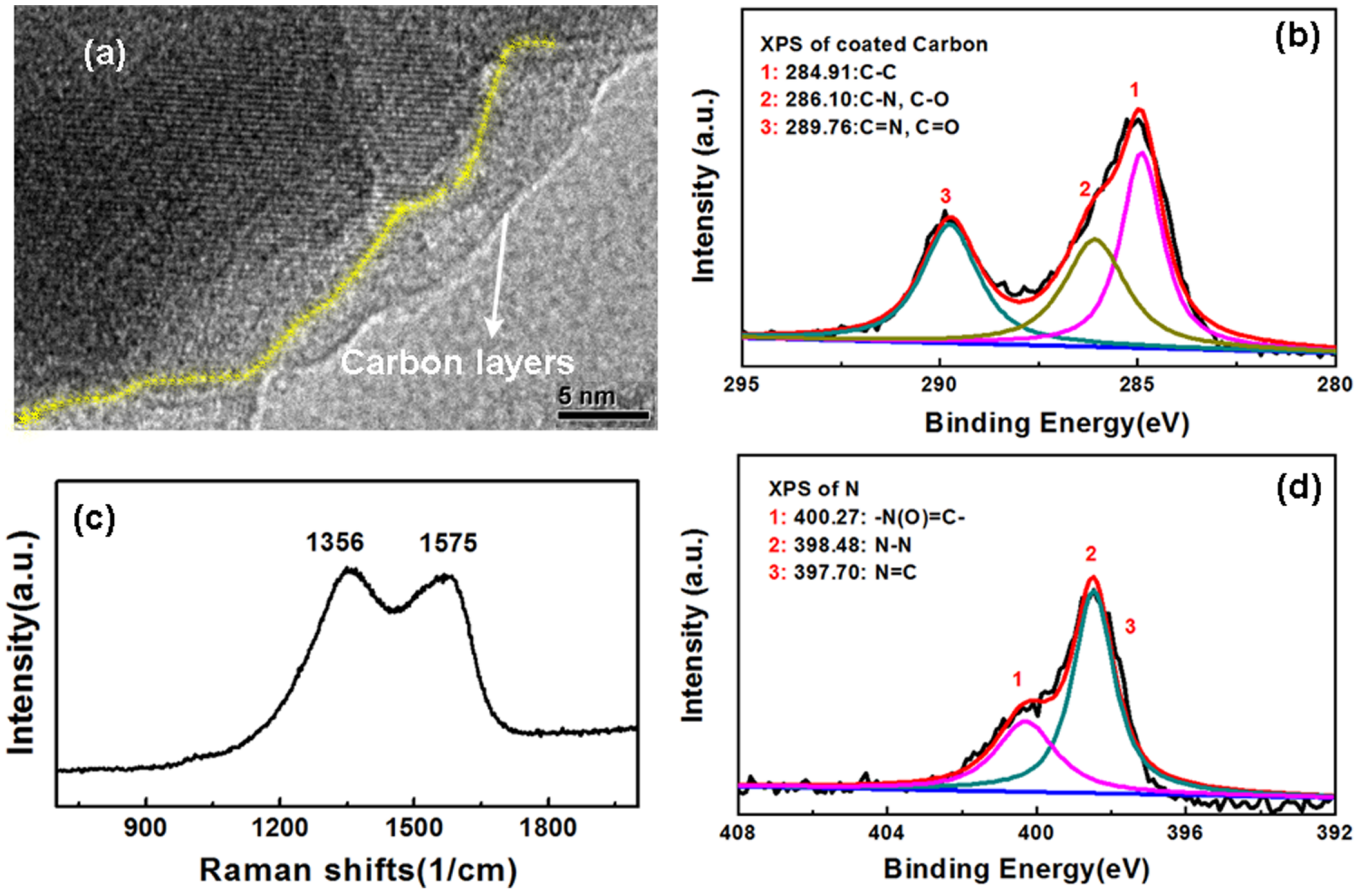
Characterization of *in-situ* nitrogen-doped carbon coated monoclinic LFS@400 material. (a) The amorphous carbon coated on the surface layer; (b) the XPS spectrum of carbon; (c) the Raman and (d) XPS spectra of nitrogen acquired from the LFS@400 sample.

The hydrothermal synthesis was conducted in the presence of organic additives, namely ethylenediamine (EN) and ethylene glycol (EG) that are known crystal growth control agents^37^,^38^. During annealing the organic additive molecules decompose resulting in coating the LFS crystals with carbon. Such *in-situ* carbon coating can be highly beneficial in the case of LFS cathode materials due to the poor intrinsic conductivity of the latter^39^. Figure 4 provides characterization data for the carbon-coated LFS@400 sample. According to the TEM images of Figure 4a and Figure S4a, the surface is distinctly covered by several nanometer thick amorphous layers, but not homogenously. By TGA analysis, the estimated carbon contents for the three LFS materials were 7.6%, 3.5% and *ca.* 0% for LFS@400 (black colored), LFS@700 (grey colored) and LFS@900 (white colored) respectively. By XPS and Raman analysis (Figure 4b and 4c), the amorphous layer was identified to be carbon speciation. The Raman peaks at 1356 cm^−1^ and 1575 cm^−1^ are the finger-prints for graphite, corresponding to the D and G bands that generally describe the interlayer and intralayer C-C vibrations in graphitized carbon. Furthermore, the carbon coating is doped with nitrogen as confirmed by XPS spectra shown in Figure 4b and 4d. Nitrogen doping is known to enhance the electronic conductivity of carbon coatings on electrode materials for Li-ion batteries^40^.

**Figure 5:**
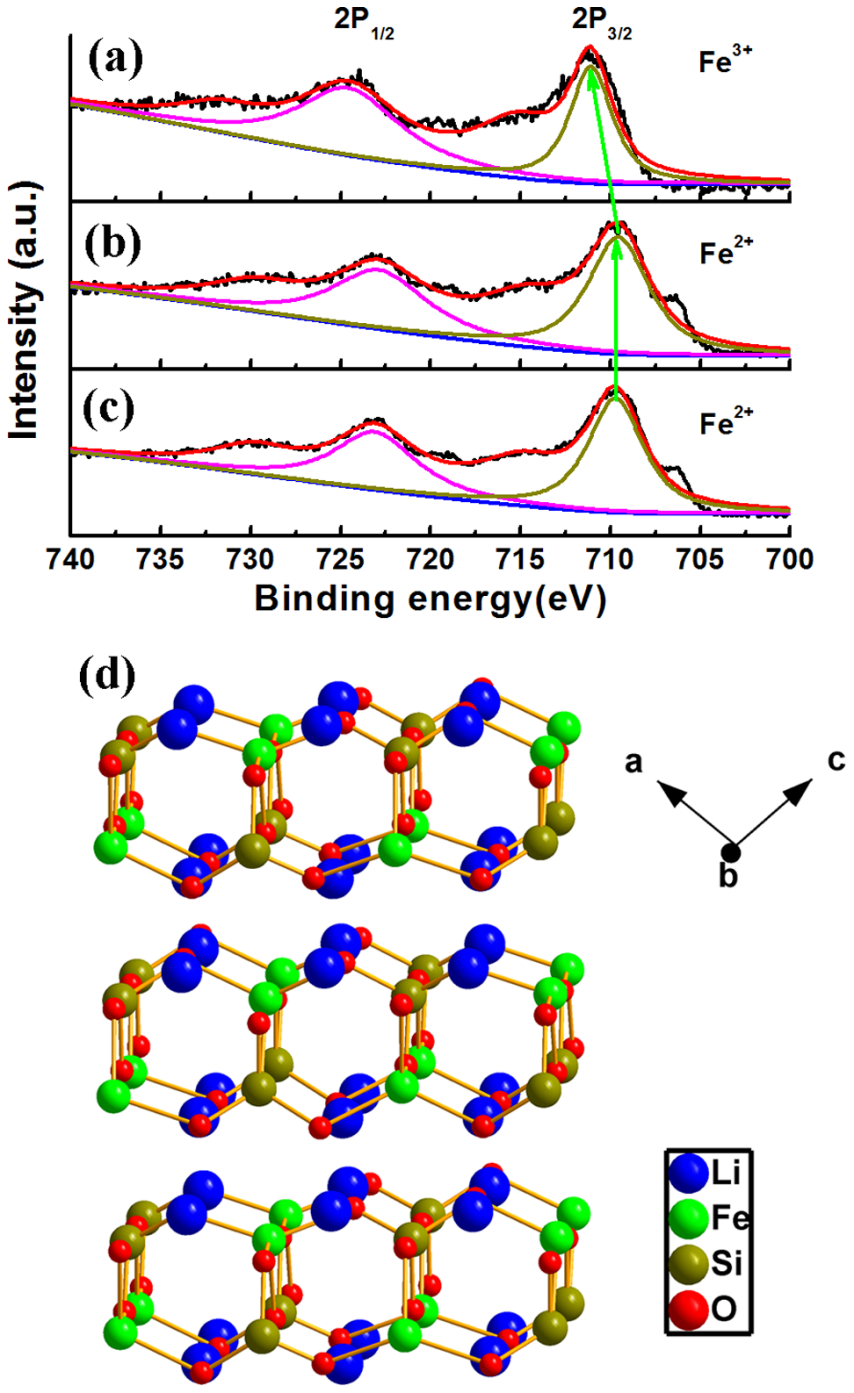
The XPS spectra for Fe in the LFS samples. (a) The XPS spectrum of Fe in the outmost surface layers of the LFS samples. Pristine LFS@400 sample only demonstrates the ferric Fe XPS signal, as is the same case of LFS@700 sample; the XPS spectra of Fe in (b) LFS@400 and (c) LFS@700 samples after 1000 V, 2 min etching; (d) schematic illustration of the monoclinic LFS (101) face.

Next XPS characterization is performed to investigate the valence of Fe ion in LFS samples. In Figure 5a, the standard ferric Fe XPS signal is detected (Fe *p_2/3_* locating at 711.08 eV) on the surface of both LFS@400 and LFS@700 samples. The presence of ferric Fe is most likely originating from surface oxidation as after etching with the argon ion under 1000 V for 2 min, a standard ferrous Fe XPS signal is only detected (Fe p_2/3_ locating at 709.30 eV). This confirms that the ferrous Fe signal comes from the Li_2_FeSiO_4_ material itself^41^. Therefore it is inferred that the as-prepared LFS@400 and LFS@700 samples is covered with thin ferric Fe contained silicates in addition to the N-doped carbon coating layer as identified by the XPS studies.

### Electrochemistry

**Figure 6:**
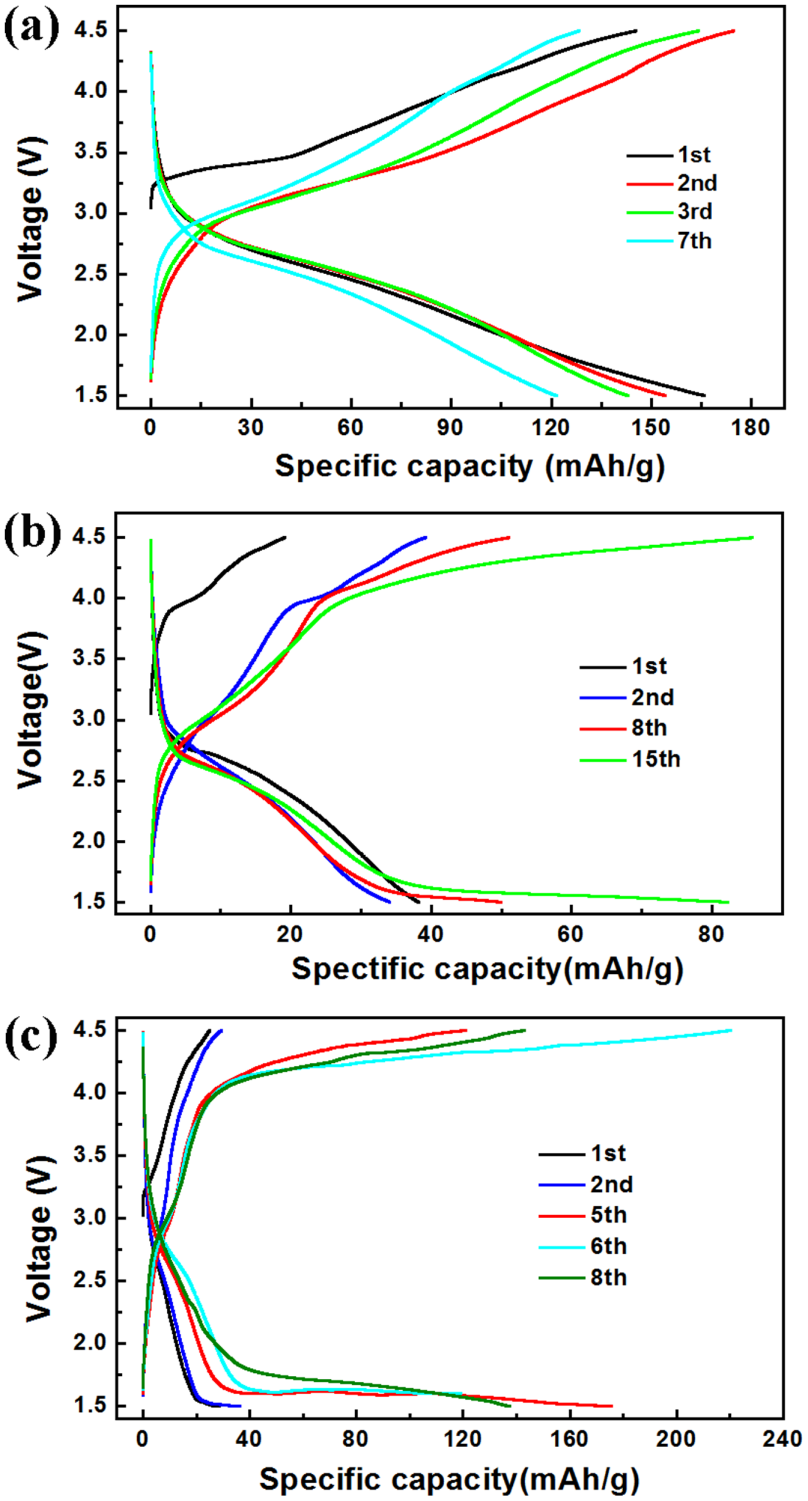
The charge/discharge performance of the pristine LFS materials. (a), (b) and (c) correspond to the LFS@400, LFS@700 and LFS@900 materials respectively cycled at the rate of C/50. The initial cycles of the LFS@700 and LFS@900 samples are also supplied in more detail in Figure S5. Note that 1C is equal to 165 mA/g.

Figure 6 presents the charge/discharge profiles of three LFS materials cycled at C/50 rate over the voltage range 1.5 to 4.5 V, where 1C is equal to 165 mA/g, *i.e.* it corresponds to 1 Li extraction/insertion from/into the host materials. As it can be seen in Figure 6a, the charge/discharge curves of the monoclinic LFS@400 polymorph exhibit typical solid-solution reaction without obvious plateaus during lithiation/delithiation. The LFS@400 electrode delivers a capacity of *ca.* 170 mAh/g in the first cycle corresponding to just over 1 Li exchange, which however fades to about 120 mAh/g after the 7^th^ cycle due to apparently increasing polarization. This large capacity loss other than arising partly from poor electronic/ionic conductivity that orthosilicate compounds suffer from^42^ can be due to structure relaxation and rearrangement involving Li-Fe interchange (or phase transitions)^23^. Actually, the Li-Fe interchange may not only influence structure stability, but also impact on the Li-ion diffusion trajectory, by which the Fe ions occupy the Li ion site in (101) plane as shown in Figure 5d, having as result to perturb/block the Li ion favorable transport paths.

By contrast to the LFS@400 monoclinic polymorph, the cycling curves for LFS@700 (mixed phase) and LFS@900 (orthorhombic phase) show remarkable differences as can be seen in Figure 6b and 6c. In this case both materials deliver very little capacity (~30 mAh/g) in the initial cycles even at the rate of C/50 as shown in Figure 6b, 6c and S5. Further cycling as per data plotted in Figure 6b and 6c does not exhibit capacity fading pointing towards a stable structure albeit associated with very low capacity. The very low capacity should be at least in part ascribed to the larger crystal size and poor electronic conductivity (no effective electronic conductivity network). Examining closer the voltage-capacity curves, firstly for the LFS@900 orthorhombic polymorph as shown in Figure 6c, it is seen the cell voltage to ascend steeply up to ~4.0 V at the beginning of the charging (delithiation) process and then attain gradually a long plateau-like profile. Then during discharging, the cell voltage moves the opposite way down to 1.6 V. Consequently, the charge/discharge process gives birth to an unacceptable high voltage polarization of about 2.70 V. By comparison the LFS@700 mixed phase material exhibits charge/discharge profile (Figure 6b) closer resembling to that of the orthorhombic LFS@900 material (Figure 6c) than the monoclinic LFS@400 (Figure 6a) despite the fact that its composition is predominantly monoclinic (90%). There are however some distinctive features. Thus in the 8^th^ cycle as shown in Figure 6b, the charging/discharging curves for LFS@700 can be divided into two parts: a slope region (*e.g.* voltage from 3.0 V to *ca.* 3.8 V of the charging curve) and a plateau-like region (*e.g.* voltage from 3.8 V to 4.5 V of the charging curve), totally different from that of the LFS@400 and LFS@900 polymorphs. This behavior can be attributed to a mixed contribution from the co-existing monoclinic phase and orthorhombic phase in the LFS@700 material.

Probing further the charging/discharging behavior of the LFS@700 material it is seen for the 15^th^ cycle as shown in Figure 6b, the plateau-like part to prolong significantly in the charge/discharge curves, a feature that provides strong indication of an electrochemically-induced structure transition from monoclinic to orthorhombic phase. However, these charge/discharge curve alterations are not obviously evident in the LFS@400 material cycled at the same rate of C/50 as shown in Figure 6a. This implies kinetic differences between the LFS@400 and LFS@700 materials when it comes to electrochemically-induced phase changes from monoclinic to orthorhombic structure. As discussed in Figures 1d, 3b, 3e, S4a and S4b, the LFS@700 sample is a mixed phase and the refinement results indicate that the monoclinic/orthorhombic ratio is approximate to 90/10 as opposed to LFS@400 that is essentially phase-pure monoclinic. It is thus proposed that the conversion of the monoclinic to the orthorhombic phase during electrochemical delithiation/lithiation becomes “catalyzed”, probably by lowering the activation energy at the monoclinic/orthorhombic interface with the minor orthorbombic component (~10%) present in the pristine LFS@700 material acting as “seed”. This finding may well explain the earlier discussed apparent contradictory phase transition results from previous studies^29^,^31^,^32^ probably arising from synthesis difficulties in obtaining phase-pure LFS polymorphs.

**Figure 7:**
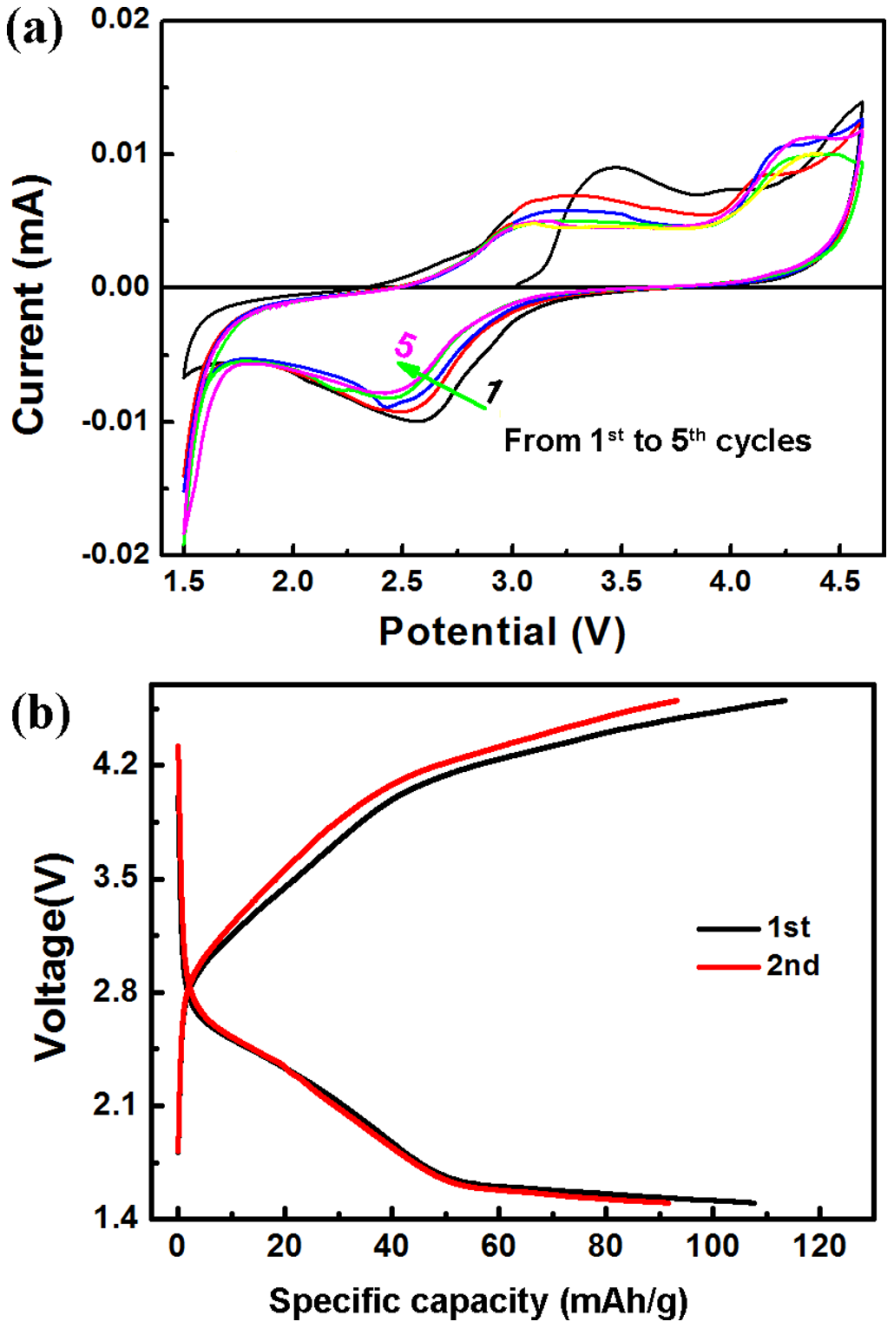
The CV and charge/discharge performance of LFS@400 material. (a) The cyclic voltammograms (CVs) of pristine LFS@400 obtained at a rate of 0.02 mV/s over the range 1.5 V to 4.6 V. (b) The charge/discharge curves of the LFS@400 material after it had been subjected to CV as in (a). The charge/discharge curves were generated at C/20 over the voltage range 1.5 to 4.6 V. Note that 1C is equal to 165 mA/g.

The phase transition from monoclinic to orthorhombic observed in the cycling of the mixed phase LFS@700 material at C/50 rate was also witnessed in the case with the monoclinic phase-pure LFS@400 material when the latter was subjected to cyclic voltammetry as revealed from the CV data plotted in Figure 7a. The CV cycles were performed under the scanning rate of 0.02 mV/s in the voltage range from 4.6 V to 1.5 V. Firstly we observe the CV profiles of the LFS@400 material not to have any sharp redox peaks, just broadened peaks with a long oxidation/reduction tail, a feature that is in accordance with the solid-solution reaction mechanism identified in Figure 6a^32^. Furthermore we observe the peak of the redox couple center at around 3.40/2.61 V in the formation cycle (black line) to shift to ~3.15/2.50 V in the subsequent cycles while simultaneously the peak intensity decreases. The high voltage oxidation peak at about 4.6 V also becomes a distributed range from 4.6 V to 4.3 V in Figure 7a. Then at the reduction process, a new peak locating at around 1.55 V appears with increasingly strong intensity in the following cycles, which is not found in the CV test under the rate of 0.1 V/s^43^. The observed changes in the CV profiles upon progressive cycling are apparently another manifestation of a complex structure evolution sequence.

**Figure 8:**
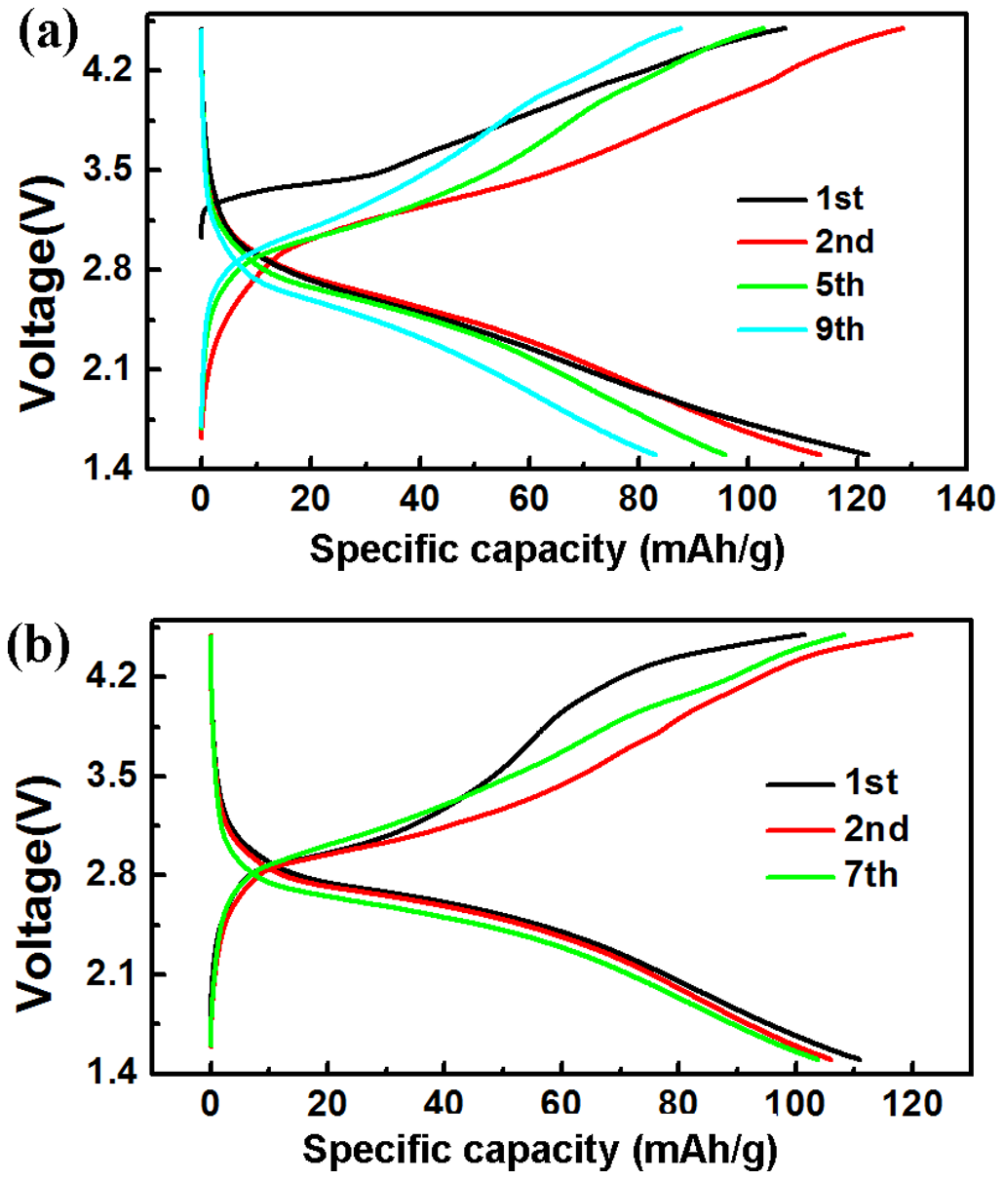
The charge/discharge performance of LFS@400 materials with different galvanostatic history. (a) Performance of pristine LFS@400 material cycled at the rate of C/20 over the voltage range 1.5 to 4.5 V. (b) Performance of LFS@400 material at C/50 over the voltage range 1.5 to 4.5 V after the cell was cycled first at the rate of C/20 for several cycles as shown in Figure 8a. Note that 1C is equal to 165 mA/g.

As to the LFS@400 monoclinic material that had been used in the 5 cycles of CV testing (at the scan rate of 0.02 mV/s in the voltage range 1.5 to 4.6 V; Figure 7a) we decided to subject it to charging/discharging at the rate of C/20 as shown in Figure 7b, because recently Masese *et al.* reported that the monoclinic LFS structure remained stable if cycled at the rate of C/20^32^. Surprisingly we found (Figure 7b) the monoclinic LFS@400 material that had been previously gone through CV testing to demonstrate nearly the same cycling profiles as the orthorhombic LFS@900 material (shown in Figure 6c). In other words here we see the mesoporous monoclinic nanocrystals (~10 nm in size) (LFS@400 material) after CV at slow scan rate to yield similar galvanostatic Li insertion/extraction characteristics as the dense orthorhombic microcrystals (300 nm in size) (LFS@900 material) despite the N-doped carbon coating of the LFS@400 material. This is powerful evidence that the original monoclinic nanocrystals (LFS@400) converted to the poorly intercalating orthorhombic phase after gone through slow cyclic voltammetry, *i.e.* slow cycling. By comparison the charging/discharging profiles of the pristine monoclinic polymorph (LFS@400) either cycled at C/50 as in in Figure 6a or at C/20 as in Figure 8a are totally different from the equivalent profiles at C/20 of the CV-tested LFS@400 material (Figure 7b). This contrasting difference is largely ascribed to the very slow rate CV tests (0.02 mV/s), which apparently allows enough time (43 hours per scan or about 18 days for the total 5 CV cycles) for transition of the monoclinic structure to take place and reach a new energetically favorable configuration, most probably the orthorhombic structure with different ionic occupations. This transition from monoclinic to orthorhombic is further verified by considering the differential capacity curves shown in Figure S7. Thus the differential capacity curve, corresponding to the 2^nd^ cycle of the LFS@400 monoclinic electrode (Figure 6a), as it can be seen in Figure S7a, manifests the broadened redox process in the whole voltage range similarly to the 1^st^ cycle of CV test depicted in Figure 7a. By contrast the differential capacity curve (shown in Figure S7b) corresponding to the 1^st^ cycle of the LFS@400 electrode that has been previously subjected to the CV scans (Figure 7a) manifests totally different redox profile equivalent to that of the LFS@900 electrode (compare Figure S7b to Figure S7c). That is, the monoclinic LFS@400 material experiences an electrochemically-induced phase transition from the monoclinic phase to the orthorhombic phase.

To investigate further the effect of rate on phase transition from monoclinic to orthorhombic during cycling we focus on the charge/discharge profiles of the LFS@400 electrode obtained at different rates and with different galvanostatic history. Thus in the case of pristine LFS@400 material upon increasing the rate from C/50 (Figure 6a) to C/20 (Figure 8a) and C/10 (Figure S6) we see the material to exhibit the same lithiation/delithiation characteristics, however with different specific capacities. Here the initial specific capacity of 170 mAh/g at C/50 (Figure 6a) is seen to decrease to 120 mAh/g at C/20 (Figure 8a) and 95 mAh/g at C/10 (Figure S6) reflecting the poor electronic/ionic conductivity properties and the tendency of structure stability for silicate cathode. At all rates we observe capacity fading after the first cycle, the extent of which seems to depend on the applied rate. Thus the capacity loss between the 2^nd^ and 6^th^/7^th^ cycles is 35, 15 and 5 mAh/g at C/50, C/20 and C/10 respectively. In other words the faster the rate the less the capacity decline suggesting less tendency for phase transition. The dependence of phase transition on applied rate and the electrochemical history of the monoclinic LFS material becomes even more clearly evident when we consider the charge/discharge data in Figure 8b. Here the LFS@400 electrode, after 10 cycles charging/discharging at C/20 rate (data in Figure 8a), was switched to the rate of C/50 for subsequent cycling (data in Figure 8b). By doing this switch we discovered the monoclinic structure to stabilize exhibiting very good capacity retention of *ca.* 110 mAh/g after several cycles (<1 mAh/g capacity loss between 2^nd^ and 7^th^ cycles). Although the pre-cycled at C/20 LFS@400 monoclinic material did not deliver as high as 170 mAh/g as the pristine material did after the formation cycle (Figure 6a) the discovery that its structure stabilized without indication of phase transition from the monoclinic to orthorhombic phase constitutes an important finding. Further structural studies are under way to fully elucidate the underlying phase transition mechanism and its dependence on the prior electrochemical material history that can pave the way to engineering orthosilicate cathode materials with full 2 Li reversible storage functionality.

## Conclusion

Monoclinic and orthorhombic polymorphs of Li_2_FeSiO_4_ have been successfully synthesized using a novel two-step process comprising organic-assisted hydrothermal precipitation followed by annealing in H_2_-Ar atmosphere. The monoclinic and orthorhombic phase Li_2_FeSiO_4_ materials are obtained by annealing at 400°C and 900°C respectively; annealing at 700°C gives birth to a mixed monoclinic/orthorhombic (90/10) phase material. The three Li_2_FeSiO_4_ materials exhibit totally different charge/discharge characteristics: the 400°C monoclinic polymorph exhibits initially one Li extraction, while the 900°C orthorhombic polymorph <0.4 Li and the mixed (90/10) phase Li_2_FeSiO_4_ material demonstrates an intermediate cycling profile. We have found the monoclinic nanocrystals to undergo phase transition to orthorhombic structure, when cycled at very low rate (C/50) resulting in significant progressive deterioration of their Li-ion storage capability. Previous cycling at very low CV scanning rate leads to hastened monoclinic to orthorhombic phase conversion. By contrast, we discovered when the monoclinic nanocrystals are cycled initially at higher rate (C/20) and subsequently subjected to low rate (C/50) cycling the intercalation performance is stabilized. The discovered rate-dependent electrochemically-induced phase transition and stabilization of lithium metal silicate structure provides a novel and potentially rewarding avenue towards the development of high capacity Li-ion cathodes.

## Exprimental section

### Material synthesis

The different phase Li_2_FeSiO_4_ nanoparticles are synthesized via preparation of an amorphous colloidal precipitate that is subsequently subjected to annealing, a method adapted from procedures developed for lithium titanate and lithium iron phosphate nanomaterials^37^,^44^. In a typical test, stoichiometric amounts (0.015 mol) of Fe(NO_3_)_3_**·**9H_2_O powder and fumed SiO_2_ powder were added into 70 mL distilled water while continuously stirring. Then a 2 ~ 5% excess of lithium acetate (CH_3_COOLi**·**2H_2_O) powder was added and the suspension became dark red. After 10 min stirring, 3.75 mL ethylene glycol (EG) was added followed by addition of 3.75 mL ethylenediamine (EN) over the next 15 min. After 30 min stirring, the dark red gel-like suspension was transferred into the autoclave and heated at 180°C for 3 hours for hydrothermal process, then at 80°C to obtain a dried amorphous precursor that was annealed in a tube furnace under a reduced N_2_/H_2 _(95:5) atmosphere. Annealing involved initially flowing the reducing gas for one hour at room temperature before the temperature was increased at 3°C/min to 200°C, where it was kept for about 2 hours. Then the temperature was increased to 400°C, 700°C and 900°C at 3°C/min for 10 hours followed by a natural cooling down to room temperature.

### Material characterization

X-ray powder diffraction (XRD) patterns were recorded in the range of 10–80° using a Philips PW 1710 X-ray diffractometer with Cu K_α_ radiation (λ ~ 1.54056 Å) and 10–110° using a Rigaku Miniflex table top XRD system using Co K_α_ radiation (1.78892 A, 40 kV; 15 mA) with a step of 0.02°, 5 sec per step at room temperature. The Rietveld refinement was performed using Fullprof software. A scanning electron microscope (Hitachi S-4700 FE-SEM) and a Philips CM200 200 kV transmission electron microscope (TEM) were employed to study sample morphology. X-ray photoelectron spectroscopy (XPS) spectra were recorded on a K-Alpha X-ray photoelectron spectrometer (Thermo Fisher Scientific Inc.). Raman spectroscopy of the samples was carried out on a Renishaw RM 3000 &InVia spectrophotometer between 500 and 3000 nm. The Brunauer–Emmett–Teller (BET) specific surface area measurements were performed using a TriStar 3000 analyzer (Micromeritics instrument corporation). Differential scanning calorimetry (DSC) and thermogravimetric analysis (TGA) was carried out on a thermogravimetric analyzer from Mettler-Toledo International Inc.

### Electrochemical tests

The discharge/charge cycling was performed using Swagelok-type cells between 1.5 V and 4.5 V or in some cases 4.6 V. Metallic lithium was used as the counter electrode. A polypropylene film (Celgard 2200) was used as the separator. The working electrode was prepared by spreading a slurry of the active material (Li_2_FeSiO_4_), conductive agent (Acetylene Black: AB), and poly(vinylidenedifluoride) (PVDF) in a weight ratio of (Li_2_FeSiO_4_/AB/PVDF) 0.70:0.20:0.10 onto aluminum foil. The each final electrode contains around 2 mg active Li_2_FeSiO_4_ materials. A standard electrolyte solution made of 1 **M** LiPF_6_/ethylene carbonate (EC)/dimethyl carbonate (DMC) (1:1 by volume) was used. The data of the discharge/charge profile was collected on an 8-Channel Battery Analyzer (MTI Corporation, USA). The cyclic voltammograms (CVs) were characterized using an electrochemical workstation (BioLogicVSP) controlled by a computer at the scanning rate of 0.02 mV/s.

## Author Contributions

G.P.D. and X.L. conceived and designed this work; X.L., H.J.W. and H.C.C. performed the material synthesis, electrochemical characterizations and prepared the TEM and SEM samples; X.L. did the analysis with R.G., P.H., A.G., K.Z. and G.P.D. assisting and advising; X.L. and G.P.D. wrote the paper.

## Supplementary Material

Supplementary Information Supplentary Info
