# Supplementary material for: Rate-dependent phase transitions in Li2FeSiO4 cathode nanocrystals
Source: Sci Rep. 2015 Feb 26;5:8599. doi: 10.1038/srep08599 (PMC4341213; doi:10.1038/srep08599)
Supplement: Supplementary Information — Supplentary Info [file srep08599-s1.pdf]

Supplementary information for:

Rate-dependent phase transitions in Li_2FeSiO_4 cathode nanocrystals

Xia Lu[1, 2], Huijing Wei[1], Hsien-Chieh Chiu[1], Raynald Gauvin[1], Pierre Hovington[2], Abdelbast Guerfi[2], Karim Zaghib[2] and George P. Demopoulos[1,*]

1. Materials Engineering, McGill University, Montréal, Québec H3A 0C5, Canada.

2. Institut de recherche d' Hydro-Québec (IREQ), Varennes, Québec J3X 1S1, Canada.

Correspondence and requests for materials should be addressed to Prof. G. P. Demopoulos:

Email: george.demopoulos@mcgill.ca ; Tel: +15143982046; Fax: +15143984492

Supporting Figures and Figure captions:

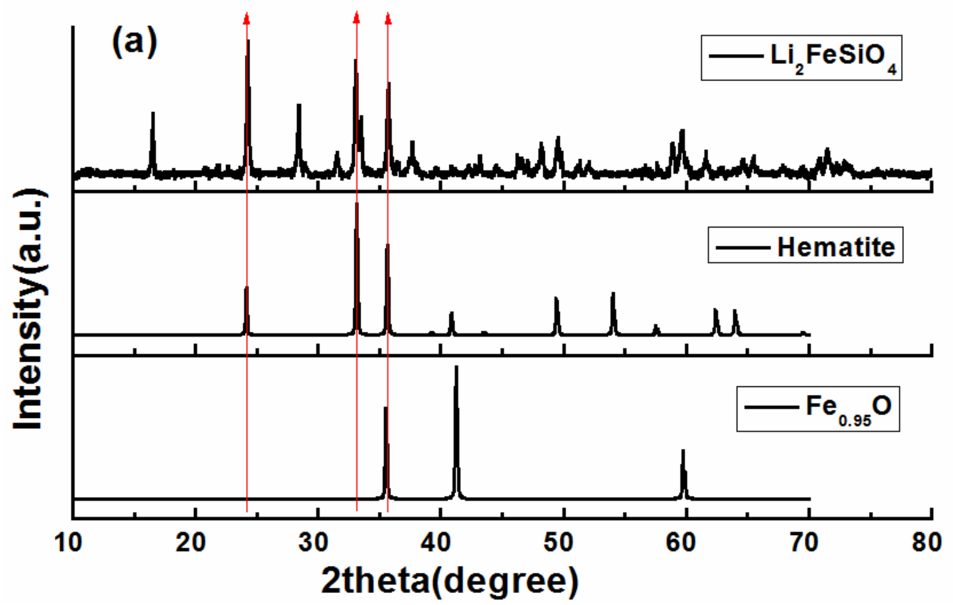

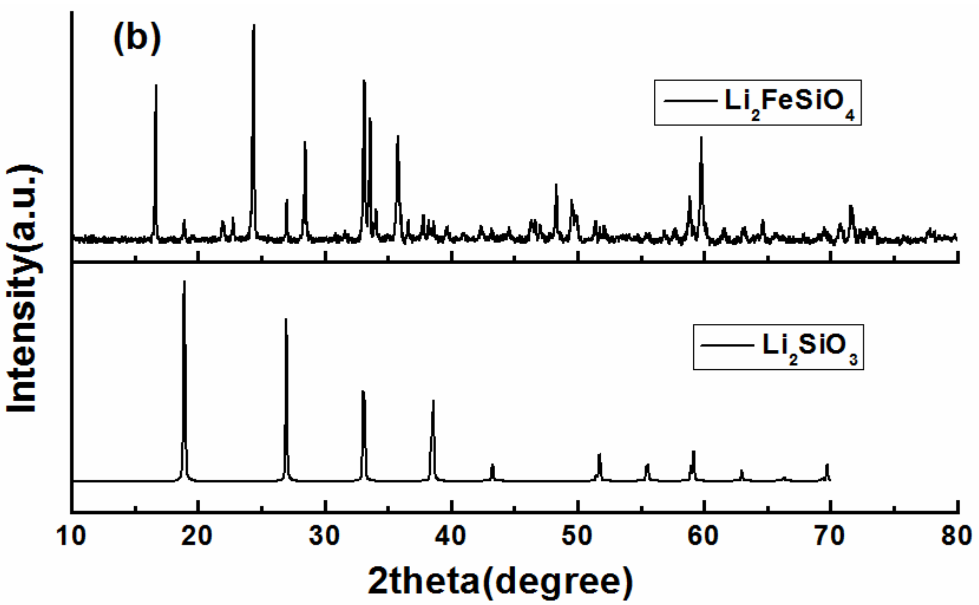

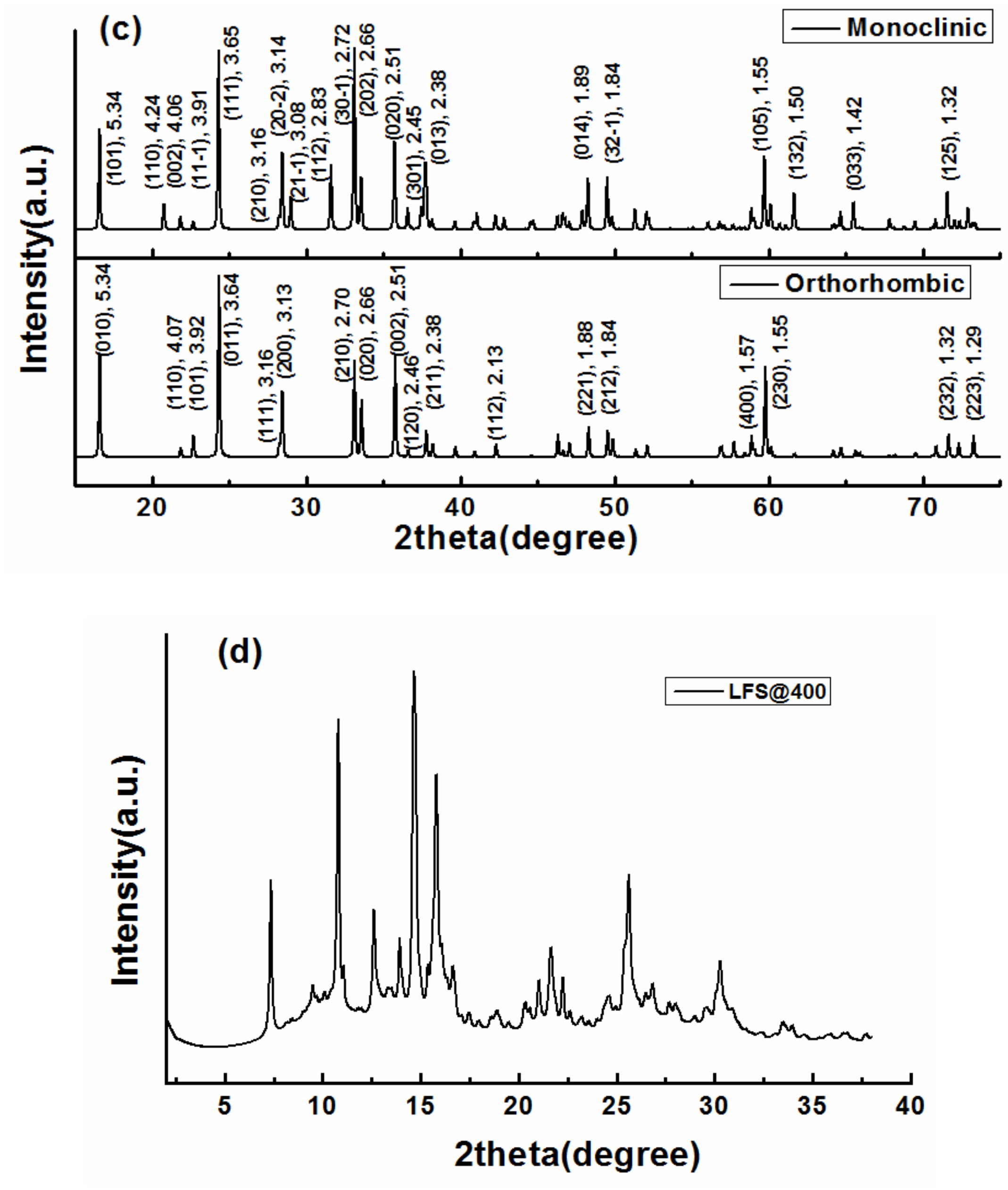

Figure S1: XRD patterns of the as-prepared Li_2FeSiO_4 samples compared to several reference patterns using Cu K_α radiation (λ ~ 1.54056 Å) source. (a) XRD of LFS@400 compared to those of iron oxides (Fe_2O_3 and $Fe_{0.95}O$); (b) XRD of LFS@900 compared to that of Li_2SiO_3; (c) standard XRD patterns for the monoclinic ($P2_1$) and orthorhombic ($Pmn2_1$) Li_2FeSiO_4 phases; (d) the XRD pattern of the LFS@400, sample (obtained at Canadian Light Source (CLS)'s synchrotron facility in collaboration with Dr. Joel Reid. The pattern was generated at 18 keV (0.68880 A) with a detector distance of 250 mm using 0.5 mm Kapton capillaries. The capillaries were subtracted from the sample patterns during integration (except for the LaB6 data, but the capillary contribution is minimal here).

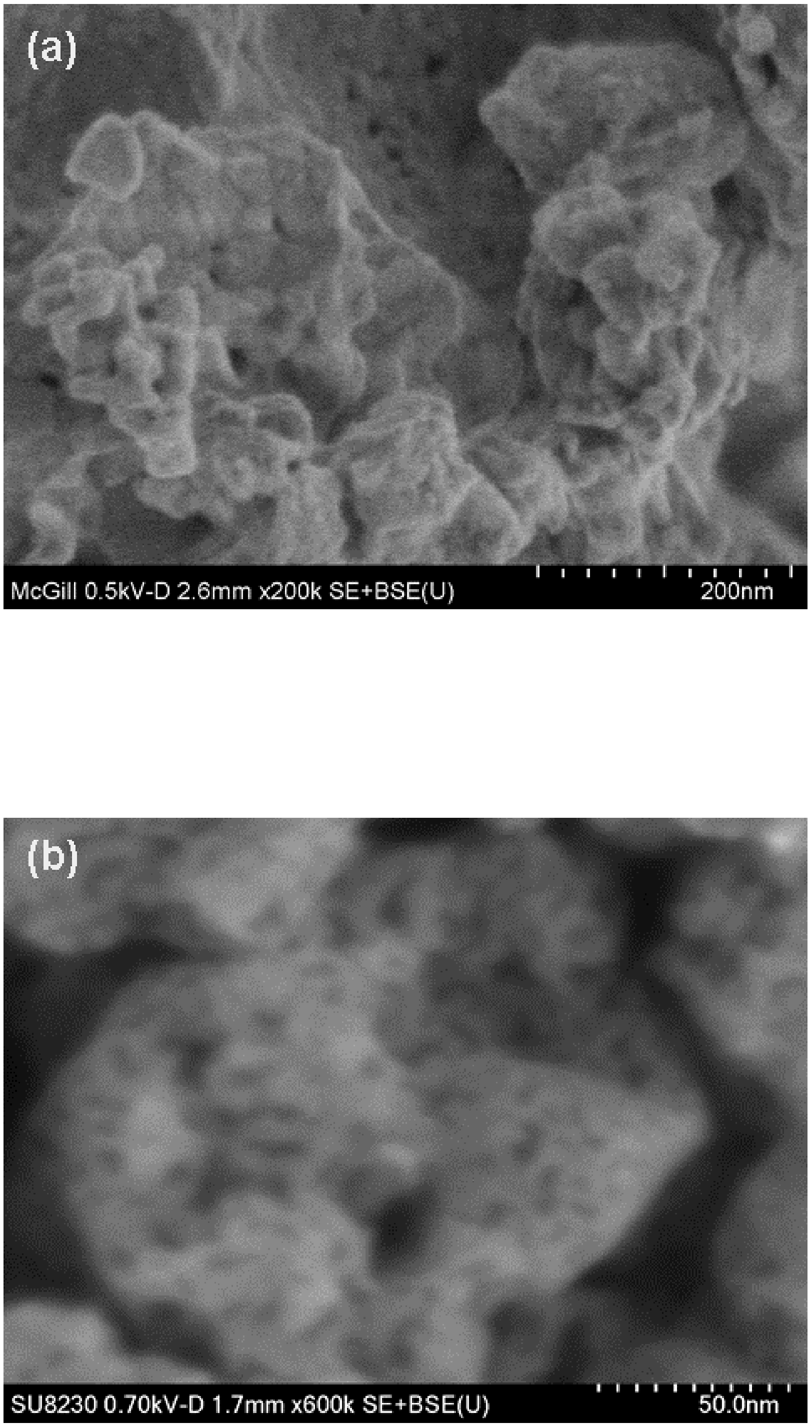
(a)
McGill 0.5kV-D 2.6mm x200k SE+BSE(U)
200nm
(b)
SU8230 0.70kV-D 1.7mm x600k SE+BSE(U)
50.0nm

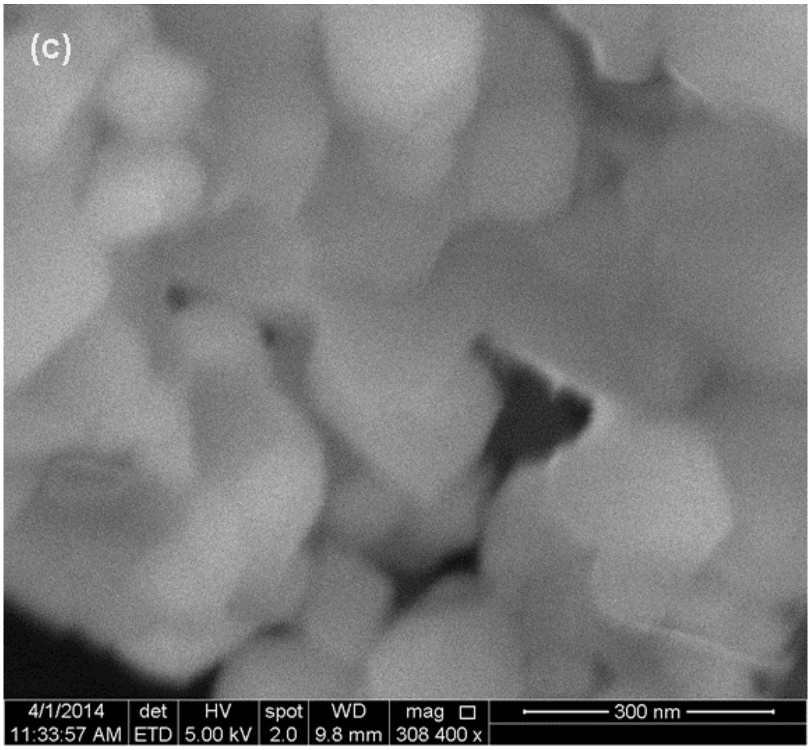

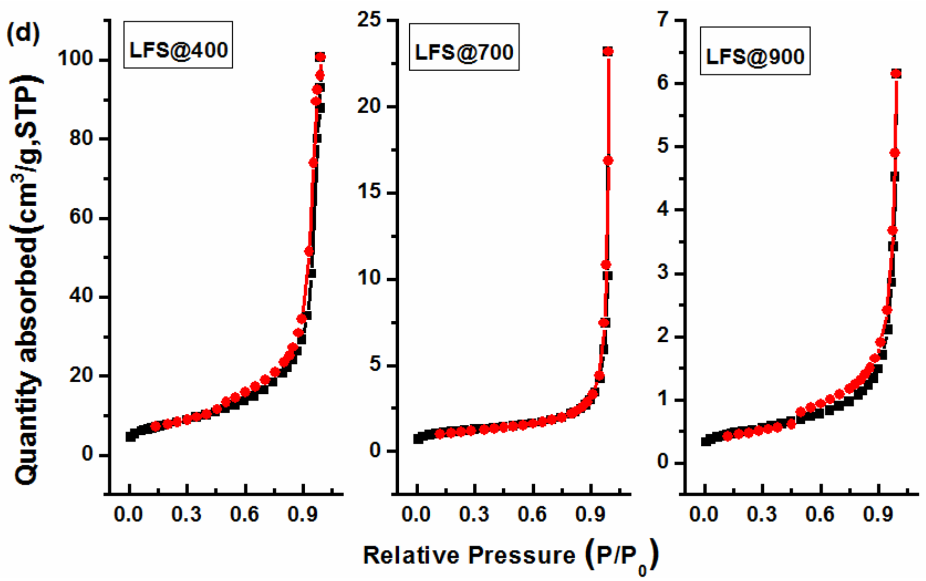

Figure S2: SEM images for the as-prepared Li_2FeSiO_4 samples after annealing at (a) 400^0C (LFS@400), (b) 700^0C (LFS@700) and (c) 900^0C (LFS@900), respectively. (d) The BET surface area plots of the three LFS samples. Complete crystal surface area and size data is provided in Table S1 and S2 below.

Table S1. The specific surface area (BET SSA) and equivalent spherical particle size data (packing factor 0.74) derived from BET specific surface area measurements (ρ=3.2 g/cm^3).

**Sample**	**BET SSA (m^2/g)**	**Particle diameter (nm)**
**LFS@400**	28.10	66.73
**LFS@700**	4.19	447.49
**LFS@900**	1.66	1129.52

Table S2. Full surface area and pore size analysis data for LFS@400 material

**Single point surface area at p/p °= 0.199489935:**	**27.06 m^2/g**
**BET Surface Area:**	28.10 m^2/g
**Adsorption average pore width (4V/A by BET):**	176.30 Å
**BJH Adsorption average pore width (4V/A):**	220.74 Å
**BJH Desorption average pore width (4V/A):**	186.56 Å

The calculated equivalent spherical particle size derived from the BET surface area (data in Table S1) is a little larger than what we observe under the microscope partly because it assumes smooth spherical shape regardless of the actual shape and roughness of the particles. The average particle size was calculated to be 66 nm, 447 nm, and 1129 nm for LFS@400, LFS@700 and LFS 900 respectively, which is close to the size observed by SEM in Figure 2.

The corresponding crystal size for the higher temperature annealed samples as determined was respectively ~ 180 nm (SEM-Fig 2b and TEM-Fig 3e) and further these crystals aggregate into a larger particle of 447 nm (BET-Figure S2d and Table S1) for LFS@700 and then ~ 350 nm crystals (TEM-Fig 3f) and 1130 nm (SEM-Fig 2c, BET- S2d and Table S1) secondary particles for LFS@900.

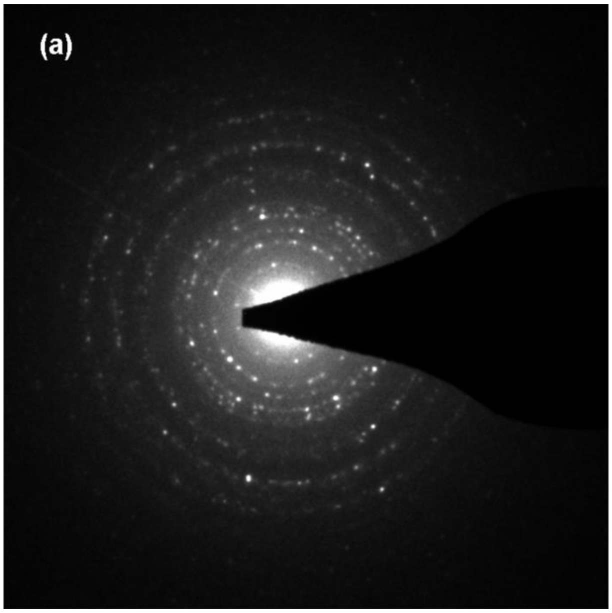
(a)

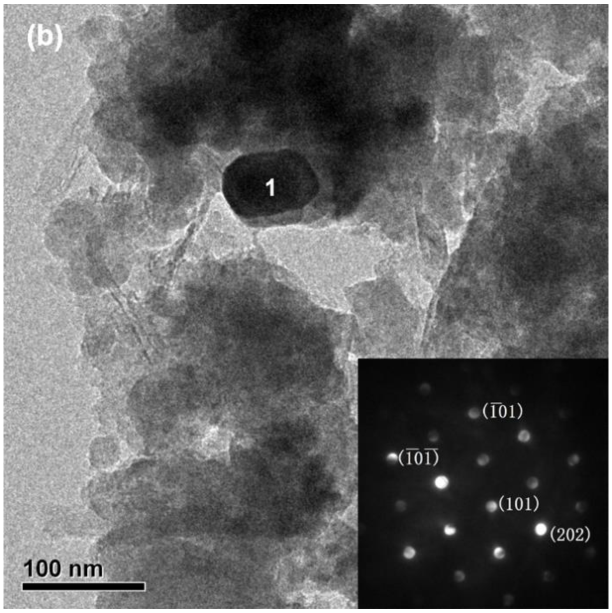
(b)
1
100 nm
($\bar{1}$01)
($\bar{1}$0$\bar{1}$)
(101)
(202)

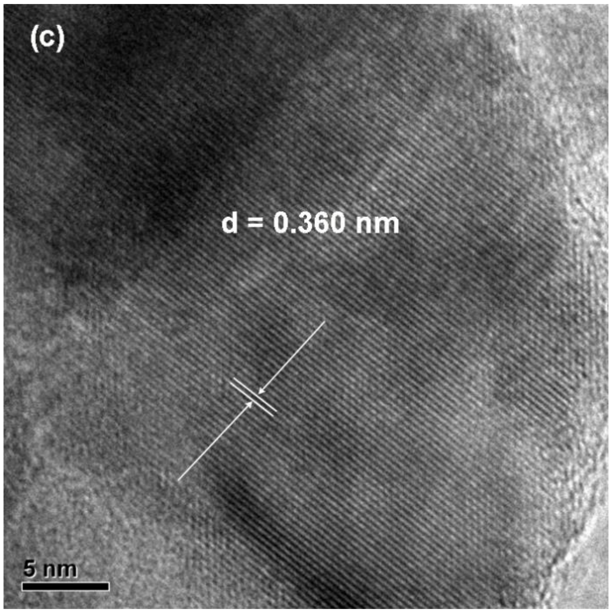
(c)
d = 0.360 nm
5 nm

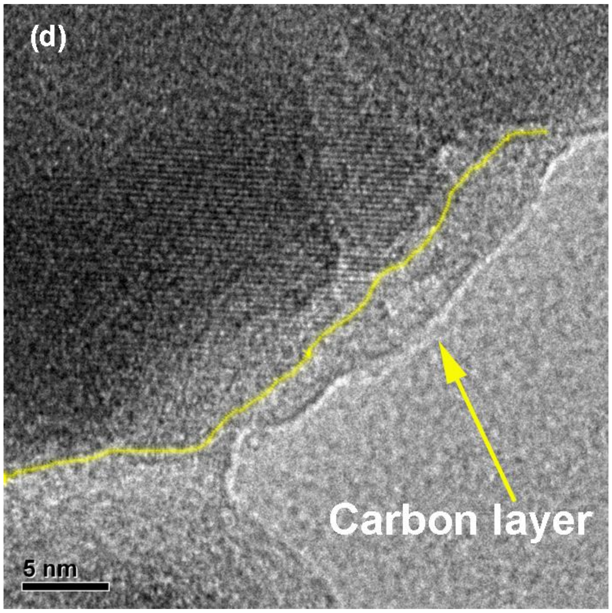
(d)
Carbon layer
5 nm

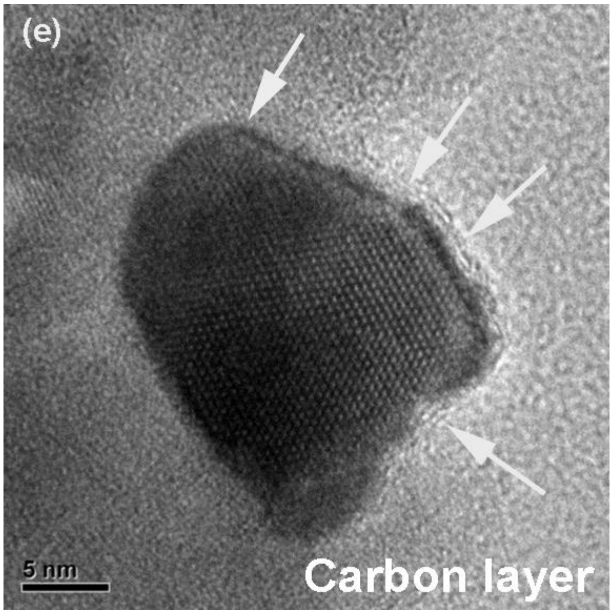

Figure S3: TEM characterization of the as-prepared LFS@400 monoclinic material obtained after annealing at 400^{0}C at the [1 $\bar{1}$ $\bar{1}$] zone axis: (a) SAED over a larger area; (b) SAED of the selected crystal 1; (c) the crystal (111) face of the monoclinic LFS polymorph; and (d) & (e) carbon-coated LFS particles.

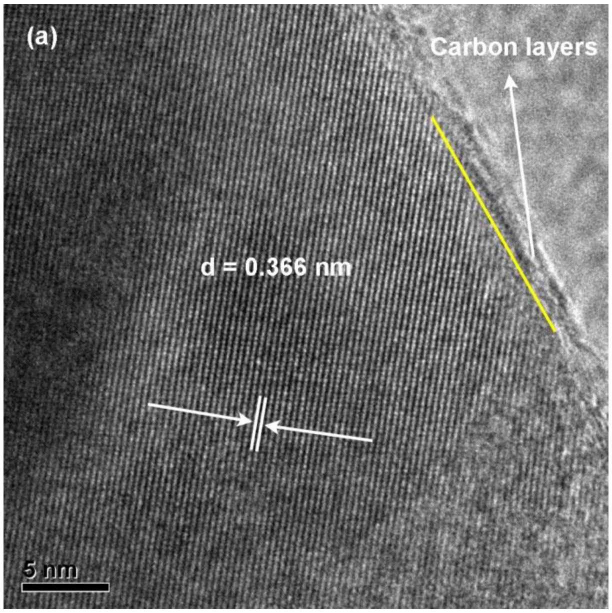

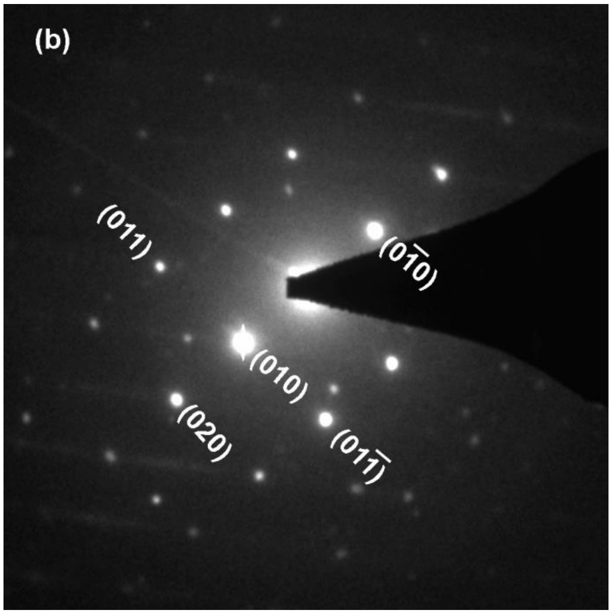

Figure S4: TEM evidence of the presence of orthorhombic Li_2FeSiO_4 crystals in the mixed phase LFS@700 material at the [100] zone axis: (a) crystal (020) plane and (b) corresponding SAED pattern.

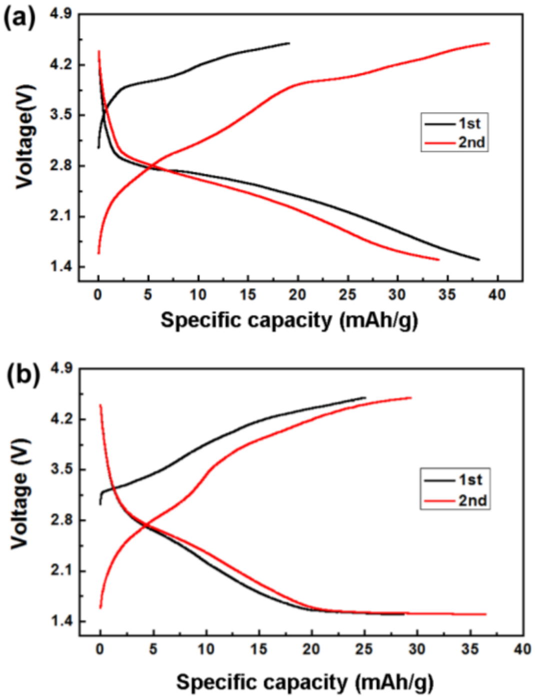

Figure S5: The initial two charge/discharge cycles of the as-prepared (pristine) LFS@700 (a) and LFS@900 (b) materials at the rate of C/50 over the voltage range 1.5 to 4.5 V. Note that 1C is equal to 165mA/g.

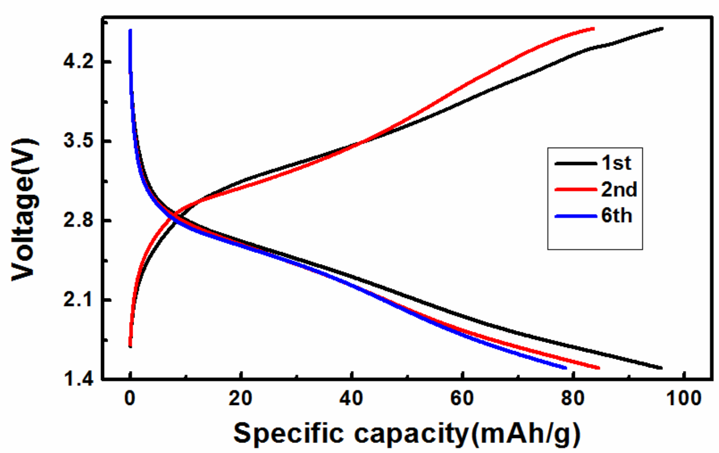

Figure S6: The charge/discharge cycles of the as-prepared (pristine) LFS@400 material at the rate of C/10 over the voltage range 1.5 to 4.5 V. Note that 1C is equal to 165 mA/g.

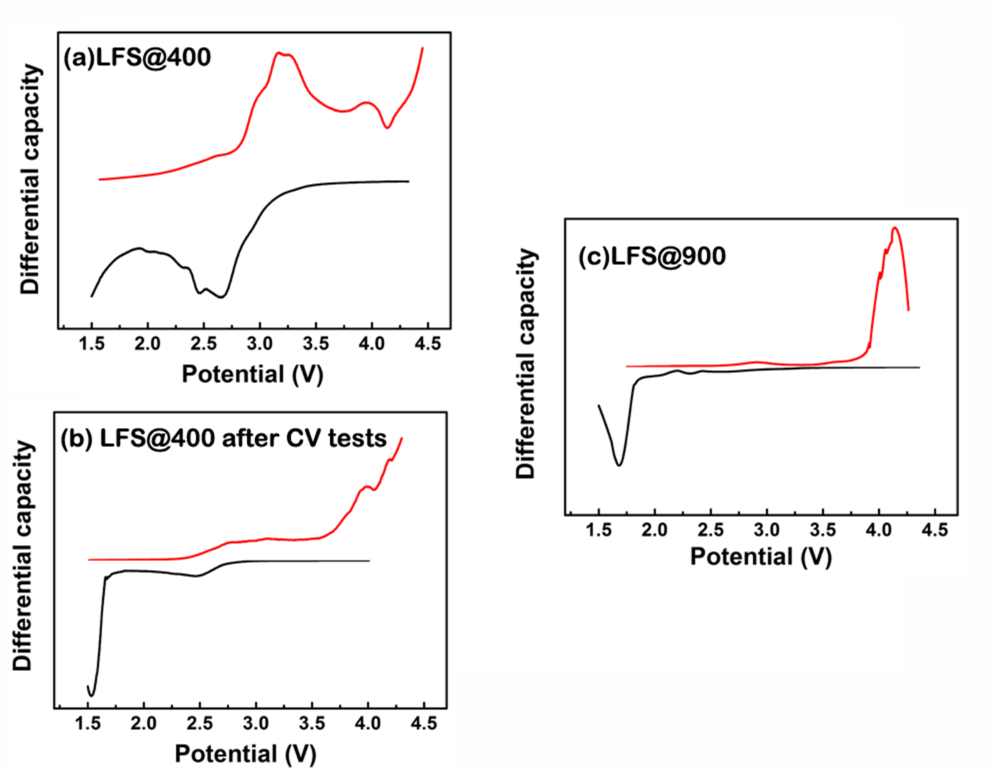

Figure S7: (a) The differential capacity curve of the 2nd cycle of LFS @400 electrode at C/50, (b) the differential capacity curve of the 1st cycle of LFS @400 electrode at C/20 after being subjected to the 5 CV scans at 0.01 mV/s (equivalent to C/43 rate; see Figure 7a), and (c) the differential capacity curve of the 6th cycle of LFS @900 electrode at C/50.
